# Genital Disorders in Children: What Does a Biopsy Bring?

**DOI:** 10.3390/dermatopathology13010012

**Published:** 2026-03-23

**Authors:** Francoise Plantier, Fiona Lewis

**Affiliations:** 1 Department of Pathology, Hôpital Cochin, AP-HP Centre-Université de Paris, 27 rue du Faubourg Saint-Jacques, 75014 Paris, France; 2 St John’s Institute of Dermatology, Guy’s & St Thomas’ NHS Trust, London SE1 7EH, UK; flewis1@nhs.net

**Keywords:** vulva, paediatric dermatology, lichen sclerosus, melanocytic naevus, atypical genital melanocytic naevus, melanoma, Crohn’s disease

## Abstract

Biopsies are only performed in less than 1% of all consultations dedicated to paediatric genital dermatology. The objectives of this paper are to review and clarify the histopathological features of the conditions most often biopsied: first, lichen sclerosus, which has a peak incidence in childhood and progresses over years; secondly, pigmented lesions, including atypical genital naevi and common naevi in the context of lichen sclerosus, both histologically differential diagnoses of melanoma, which probably does not present in childhood. And finally, Crohn’s disease, which is a cause of vulval oedema or genital ulceration.

## Simple Summary

A genital biopsy is rarely needed in children, as the common conditions usually have clear clinical features. Pigmented lesions and atypical presentations of disease may need expert histological examination. This paper discusses these scenarios, covering diagnostic difficulties and clinico-pathological correlation.

## 1. Introduction

It is rarely necessary to biopsy an inflammatory dermatosis or vulval lesion in a child and is only performed in less than 1% of all consultations dedicated to paediatric genital dermatology [1]. The common conditions seen in this group are lichen sclerosus, irritant dermatitis and psoriasis. These are generally diagnosed on the basis of their clear clinical features, and therefore a biopsy is not required, which may need a general anaesthetic in a young child. However, in cases where the features are atypical, histology can be helpful. Biopsies are performed in cases of clinically atypical lichen sclerosus (LS), atypical pigmented lesions, or less common lesions such as syringomata and inflammatory linear verrucous epidermal naevus (ILVEN).

Syringomas are benign eccrine sweat gland tumours and present as small nodular lesions that can be itchy. There may also be lesions on the face. If syringomatas are not clinically typical, they are always pathologically easy to diagnose. They have the typical epithelial duct structures in the upper dermis, which have a very characteristic ‘comma’ or ‘tadpole’ appearance. ILVEN has no histological peculiarities at a young age in this location. The features are those of psoriasiform hyperplasia with alternating parakeratosis and orthokeratosis. The differential diagnoses to ILVEN are candidiasis, flexural psoriasis, and a CHILD-Naevus, especially interesting as a differential diagnosis in children, because of the genetic implications. The CHILD (congenital hemidysplasia with ichthyosiform erythroderma and limb defects) syndrome is an X-linked dominant condition that mainly affects females and is generally lethal in males. It is due to a defect in the NSDHL gene affecting cholesterol metabolism. Recognising the CHILD naevus is important because of the associated problems.

The difficult lesions with diagnostic pitfalls and differential diagnosis relate to LS and melanocytic proliferations, and these will be discussed. We will also briefly mention Crohn’s disease, where biopsy can be helpful in cases of isolated genital oedema or ulceration.

### 1.1. Lichen Sclerosus

LS is one of the most common reasons for a vulval biopsy to be performed in women presenting with discomfort, pruritus or changes in the normal vulval anatomy. The characteristic clinical features in girls and adults include white sclerotic plaques, which are usually symmetrical and affect the inner labia majora, labia minora and clitoral hood. The pathognomonic feature of LS is ecchymosis or purpura, which can be particularly marked in children and is often due to scratching and rupture of small dermal vessels. If untreated, the inflammatory disease can alter the normal vulval anatomy, leading to resorption of the labia minora, sealing of the clitoral hood over the glans clitoris and introital narrowing due to labial adhesions. The vagina is unaffected.

In males, it is a common cause of phimosis. It is predominantly a disease of adults, with approximately 5–15% cases occurring in children. The incidence of paediatric LS in Sweden is 80.9 per 100,000 persons per year, with a higher incidence in females (114.4) than in males (47.2) [2]. In a series of 130 prepubertal girls with vulval complaints, 18% were found to have LS compared with 33% having atopic or irritant dermatitis, and 17% psoriasis [1].

In circumcision specimens, signs of LS are found in about 32% of the cases in adults [3] and between 10 and 83% in children, with or without phimosis [4]. The incidence in males has been difficult to establish, as previously, circumcision specimens were not always subjected to histological examination.

Vulval LS can occur at any age, although the highest incidence can be observed in women aged 40–60 years old, and in pre-pubertal girls, with a clear peak of incidence in girls aged 4 to 6 years. This represents 7–15% of all vulval LS cases [2,5,6]. In boys, some authors say there is a peak incidence at the ages of 9 to 11, which is later than in girls [7], whereas in other studies, the bimodal age distribution is not observed in the male population, where a single peak in incidence emerges prominently around 20 years of age, perhaps because of an underdiagnosis of mild LS.

LS is almost never biopsied in boys and only observed on posthectomies [8], and rarely biopsied in girls, except if clinically atypical, to rule out juvenile pemphigoid.

A biopsy should not be required to differentiate between LS and vitiligo, as this should be a clinical diagnosis. The skin may be pale, but the texture will be normal in vitiligo, and there should be no symptoms. Clinically, there can be difficulties when LS and vitiligo occur together, as can occur in children [9].

**Figure 1:**
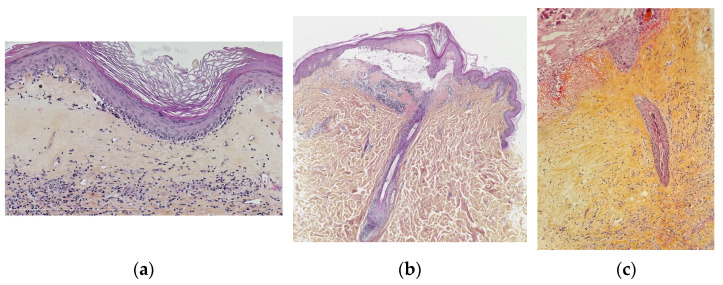
Lichen sclerosus. (**a**) Typical lichen sclerosus: hyperorthokeratosis, epithelial atrophy, dermal superficial sclerosis containing melanophages, band-like lymphocytic infiltrate under the sclerosis. (**b**) Perifollicular lichen sclerosis: superficial sclerosis visible only around the ostium of a follicule and with underlying lymphocytic infiltrate. (**c**) Syringitis in lichen sclerosus: acrosyringeal acanthosis, and the influx of neutrophils) into the sweat gland epithelium and basal membrane thickening.

Vulval LS in children shows the same characteristics as described in adults, i.e., vacuolar interface dermatitis with an atrophic epidermis, band-like dermal fibrosis and lymphocytic infiltrate. The most constant signs, found in more than 90%, are hyperkeratosis, low lymphocytic exocytosis, vacuolization of the basal keratinocytes and dermal superficial sclerosis with hyalinized collagen. In 40 to 90% of the cases, epidermal atrophy, basal apoptotic keratinocytes, a band-like lymphocytic infiltrate (moderate to severe), interstitial infiltrate, ectatic vessels, and melanophages may be seen (Figure 1a). Surprisingly, basement membrane thickening and hyalinized vessels are rare (8 and 23%, respectively), while it is very common in adult vulval LS, perhaps being a long-term effect. One finding, not well documented in the literature, that could be a peculiarity of paediatric vulval LS is periappendageal and, in particular, perifollicular dermatitis with lymphocytic exocytosis, vacuolization of keratinocytes, apoptotic keratinocytes at the hair follicle, sometimes with follicular acanthosis and basal membrane thickening. However, there is also lymphocytic syringotropism, acrosyringeal acanthosis, and the influx of neutrophils (neutrophilic syringotropism) into the sweat gland epithelium and basal membrane thickening (Figure 1a–c) [7,10].

Statistically significant differences have been noted for some characteristics between infancy and the teenage years. Lymphocytic exocytosis of the epidermis is more important, and melanophages in the dermis and hyalinized sclerotic vessels are present more frequently in biopsies after puberty than those taken from girls under the age of 12, perhaps because of the longer duration of the inflammation [10].

While pruritus due to LS is frequent in girls, in young boys, LS is often silent, there are no functional difficulties, and the white lesions are often ignored. Without treatment, balano-preputial synechiae, phimosis and urethral stenosis may require circumcision [11], and it is usually the urinary symptoms that lead to the diagnosis. Biopsies taken from the penile foreskin in boys with LS are similar to those taken from the vulva. The typical histological features in long-standing LS are hyalinised, homogenised collagen and pseudo-oedema in the upper dermis, a lichenoid band in the mid dermis, and orthokeratotic hyperkeratosis in the epidermis. In more advanced LS, epidermal atrophy is seen, but there is an absence of lichenoid inflammation. However, there can be different patterns at different sites in the foreskin with established lesions in the mucosal surface (inner preputial surface) and early LS at the mucocutaneous junction (tip). In early LS, the epidermis is normal with or without focal widening of the basement membrane. The outer preputial skin may be histologically normal. This pattern of involvement of the inner preputial surface with sparing of the outer surface would indicate that the inner surface is the first area to be involved when the disease starts. Singh found that a cleft in the inner preputial surface is a very common finding (87%) in well-established LS in males [10]. It is not a true dermoepidermal bulla but is due to a lack of cohesion of the superficial dermis, perhaps because of lymphoedema. Lymphocytic phlebitis, rarely described before, is reported in about 10% of cases [10].

The changes caused by LS, which may be mistaken for early LS, must be differentiated from other causes of secondary phimosis, mainly nonspecific chronic inflammation secondary to previous episodes of trauma (accidental/iatrogenic) or infection. Poor hygiene can lead to chronic inflammation and then phimosis. In these cases, the inflammation is frequently associated with a fibrosis which is not hyaline or homogenised at all, but made up of thick collagen bundles, with pericapillary inflammatory infiltrates, rich in plasma cells around capillaries, often vertical in orientation.

Lichen planus (LP) is another differential diagnosis and may be difficult to differentiate from LS, especially in the early stages of LS. In LP, the infiltrate is adherent to the basement membrane, but this feature may be observed in LS with superadded inflammation. The best sign of LS is the hyaline band in the superficial dermis with the disappearance of elastic fibres. Genital lichen planus is generally extremely rare in children, and most reports are in Middle Eastern or Indian populations.

In some cases, the histologic differences between LS, LP and pemphigoid are not that clear and may overlap. A subepidermal split may be observed in LS or LP, but this is rare. This would correlate clinically with the bullous form of LS, which is sometimes seen in children. It is due to the vacuolar degeneration of the membrane zone and/or the keratinocytic monocellular necrosis, which are absent in pemphigoid, where the basal layer is normal, basaloid and laid out in a palisade [12].

Malignant transformation to squamous cell carcinoma has never been reported in male paediatric LS [7]. In a study of 328 biopsy-proven cases of LS in females up to 18 years of age, no cases of dysplasia or suspicion of a premalignant lesion were seen [10]. One study suggests that a longer duration of adult-onset disease can increase the risk of a squamous cell carcinoma [13]. However, this does not seem to be the case for those who have pre-pubertal disease. Studies following children and adolescents with vulval LS into adulthood are needed to better understand the course of this disease and its repercussions [10]. In many cases of well-treated disease in childhood, vulval LS will improve at puberty and resolve with no further problems. However, some will continue to have symptoms and require ongoing treatment into adult life.

### 1.2. Pigmented Lesions in Children

The prevalence of pigmented vulval lesions in children or teenagers is uncertain. In a series of more than 1000 children and adolescents evaluated for naevi, the prevalence of genital naevi was 3–5%, with a male:female ratio of 1.3:1, without any statistically significant differences in age, sex, total number of naevi, or family history of atypical naevi and melanoma. A globular dermoscopic pattern was observed in 93%. The onset of genital naevus was before the age of 2 years in 67% of patients. Most genital naevi underwent a gradual change in diameter, elevation, colour and texture, or a combination of these factors with age [14]. Lentigines may affect the genital skin in isolation or occasionally as part of a syndrome such as LAMB (lentigines, cardiac myxomas and multiple blue naevi). These can be diagnosed on clinical and dermoscopic features and are not discussed further here.

Genital naevi in children often worry parents and physicians as they can have very atypical features and are often very dark. Although dermoscopy and newer diagnostic tools such as confocal microscopy may help, the gold standard of diagnosis is histological examination. Changes in genital naevi are more difficult for patients to monitor than naevi at other sites, as they are not so easy to see, especially in females. If a biopsy is felt necessary, this should be performed under general anaesthesia to reduce the trauma of the procedure, especially in younger children. The lesion does not need wide local excision initially, and this should reduce scarring, which can be a significant issue in specific areas such as the clitoral hood [14]. Vulval melanoma under the age of 40 is very rare and is exceptional in children. The rare published cases of paediatric vulval melanoma are often disputed and may well have been atypical genital naevi.

There are two diagnostic pitfalls in melanocytic vulval pathology that may lead to a false diagnosis of malignancy: the existence of atypical genital naevi, especially in young people, and the possible coexistence between LS and a melanocytic lesion.

## 2. Histologically Atypical-Looking Genital Naevi

It is well demonstrated that naevi in certain locations, such as the genitalia and breast, have atypical clinical and histological features and belong to the group of naevi with special-site features. On the genitalia, they are called “atypical genital nevi (AGN)” [15]. They are often dark, with an irregular border, and can look alarming. Histologically, they hey are mostly compound melanocytic lesions, well-demarcated and symmetrical. They may be large, very cellular, and deeply pigmented.

**Figure 2:**
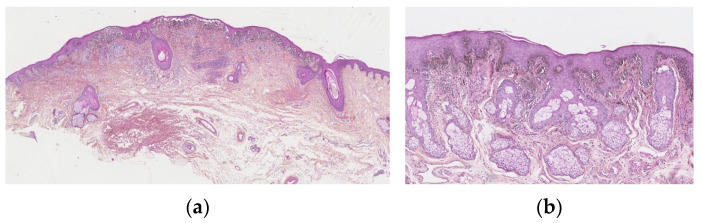
Atypical genital nevus. (**a**) Florid junctional component. Nests that show prominent retraction artifacts, are confluent, dyscohesive. Dense derman component with fibrosis. (**b**) Lentiginous epidermal component, bulky, epithelioid polygonal melanocytes with scant cytoplasm and angulated hyperchromatic nuclei, or multinucleated.

The junctional component is usually florid. They commonly show a mixture of several patterns. Most are in a nested pattern, where larger but variably sized, oval nests are predominant and can be oriented perpendicular and parallel to the dermo-epidermal junction. The dyshesive pattern shows discohesive nests, which may be almost contiguous, forming a band that separates the dermis from the epidermis. A *crowded pattern* is rarely seen with ill-defined nests and single melanocytes, which obscure the dermo-epidermal junction. The cellular nests may show prominent retraction artefacts. They can be irregularly distributed along the epidermal rete ridges, but sometimes confluent, with a parallel alignment to the dermo-epidermal junction. A huge lentiginous component can be seen. Pagetoid spread into the granular cell may be observed, but only focally and in the centre of the lesion. Adnexal involvement is frequent (Figure 2a,b).

In summary, melanocytic atypia of the junctional component is typically present. In contrast to dysplastic naevi, melanocyte atypia is uniform, rather than random. Melanocytes may be epithelioid (without any other criteria of Spitz lesions), polygonal with scant cytoplasm and angulated hyperchromatic nuclei, or multinucleated. Infrequently, they have a spindle cell morphology. The epidermis may be acanthotic or atrophic.

The dermal component is often prominent and mushroom-shaped. It may display the same cytologic atypia seen in the epidermis, but there is no deep dermal or atypical mitosis, and there is a cytologic “maturation” visible. Broad bands of fibrosis may be visible in the superficial dermis. Some authors have suggested that all these lesions need excision, and this is often performed in adults. However, in children, where there is no change and there are normal dermoscopic features, follow-up with clinical photographs and dermoscopy is a very reasonable approach [15].

Morrel from the Dutch Pathology Registry database identified 29 cases of such atypical vulval naevi, i.e., 4–6% of all vulval naevi in young people. They have no clinical peculiarities and cannot be clinically distinguished from ordinary naevi [16]. In another study, Clark found that of 56 vulval lesions biopsied for suspicion of a melanoma, AGN represented 1/3 of the cases [17]. This must be kept in mind in interpreting any vulval pigmented lesion, as it is important to avoid wide local excision, which can result in significant scarring and psychological trauma.

Regarding other types of nevi, many varieties have been described in the genital area, but they are all rare. These include Spitz nevus, agminated Spitz nevus, blue naevus, balloon cell naevus, cellular blue naevus, desmoplastic naevus, halo naevus, BAPoma and deep penetrating naevus. There is no data on the use of immunohistochemistry or PRAME in the paediatric population with genital naevi.

## 3. Naevi in the Context of LS

In the context of LS, naevi can appear histologically very atypical. Post-inflammatory pigmentation is frequent in vulval LS, which is one of the most frequent causes of multiple vulval pigmented lesions in adults, mainly because of the number of melanophages. Their presence in the superficial chorion testifies to the alterations of the basal keratinocytes and the resulting melanin incontinence. However, pigmentation secondary to LS is much less common in children. In a study about histopathological characteristics of vulval LS in a juvenile population, Morrel described melanophages in the dermis in about 40% of the cases, but it is not correlated with clinical findings [10].

LS can occur without symptoms and a naevus may be ignored and discovered only when LS clinically manifests itself, and conversely, LS may be discovered on the excision specimen of a pigmented vulval lesion. Moreover, a naevus may be modified by scratching, and pruritus is a frequent symptom of LS.

**Figure 3:**
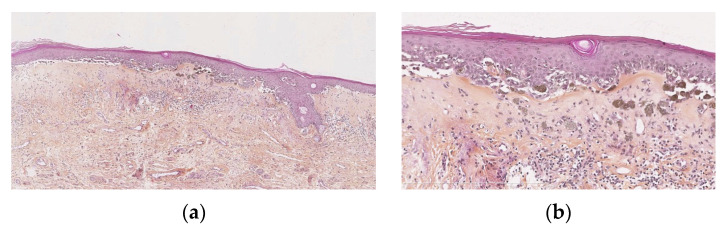
(**a**) Junctional naevus in the context of lichen sclerosus: band-like superficial fibrosis, epidermis showing a tendancy to atrophy and ll-defined, irregularly pigmented lesion. (**b**) Lentiginous epidermal component, dyscohesive, confluent, ill-defined epidermal nests, melanophores in the superficial dermis.

Melanocytic proliferations with LS are rare and are histologically difficult to interpret [18]. They have features in common with persistent nevi, also called recurrent nevi or pseudomelanomas, that are recurrences of pigmentation that appear after incomplete removal of a nevus and can mimic malignant melanoma [19,20]. They can also present with criteria that are those of atypical naevi (Figure 3a,b). Morrel compared 5 biopsies from naevi without LS and 12 with LS: cytological atypia was seen in 83% versus that seen in 40% without LS. The same author, from the Dutch Pathology Registry database, identified 16 cases of atypical naevi in the context of LS, coming to the conclusion that 36% of all nevi with LS are atypical, versus 4.6% of all vulval naevi in young people.

The lymphocytic inflammation in LS may contribute to cytological atypia in melanocytes [10].

In conclusion, there must be a clinico-pathological correlation, and the pathologist must ask clinicians if there are clinical signs of LS, and also always keep this in mind as a possibility, even if LS is not obvious in the sample.

## 4. Genital Melanoma in Children?

Cutaneous melanoma is rare in the paediatric population, but there is little robust epidemiological data. Incidence and mortality rates were estimated at 0.13 and 0.02 per 100,000 population, respectively [21]. In the United States, the incidence rate of paediatric melanoma (PM) is estimated as 5–6 per one million children and adolescents, but there are conflicting reports of any trend in incidence [22,23]. In the published series of paediatric melanoma, it is difficult to distinguish the proportion of melanoma in the genital area, the details about locations being no more accurate than “head/neck/trunk/limbs” [24,25].

There are only two published cases of genital melanoma in boys, and one case of a malignant melanoma developing from a giant congenital naevus involving the penis [26].

As the mean age of vulval melanoma is 54–76 years, it may be questioned whether a genital melanoma at a young age exists at all. In a Dutch study, no cases of vulval melanoma were recorded under the age of 18 years over a 30-year period from 1991 to 2020. No melanoma developed after a vulval naevus either [16]. Hunt reviewed charts of 1159 children given the diagnosis of genital melanocytic naevi: no melanoma was observed in the follow-up, but it was very short (1.5 years) [14].

There are nevertheless a few published cases of vulval melanoma under the age of 18, but a number of these cases were challenged. Considering that the majority of the published cases of juvenile vulval melanomas are described in the context of LS, there is great doubt about whether these are true melanomas, because of the reasons previously discussed. No recurrence or metastasis has ever been described in the published cases. There is one case of a positive lymph node in a 10-year-old girl [27]. In a large study of 1463 cases of vulval or vaginal melanoma from 1972 to 2010, only 13 cases were between 10 and 19 years of age. The 10-year survival was 100%, which again casts doubt on the true diagnosis [28]. There is no literature that reports recurrence or mortality during follow-up, again questioning the true diagnosis.

In the past, clinicians have been encouraged to biopsy and excise pigmented vulval lesions in children to rule out melanoma. However, the large Dutch studies show that a vulval naevus in the paediatric group is highly unlikely to be a melanoma. Therefore, full excision is not indicated if there are features of atypical genital naevus on any incisional biopsy [16].

## 5. Crohn’s Disease

Crohn’s disease can present in childhood and should always be considered in the differential diagnosis of vulval or penile oedema, nodules, ulceration and fissuring [29,30]. It can present at the same time as the gastrointestinal symptoms but may occur in isolation in the ano-genital skin before any inflammatory bowel disease symptoms occur. It is therefore very important to recognise it at this stage, as there are often long delays in the diagnosis of the condition in the skin and in the bowel.

Histology would be the same as that seen in adults with non-necrotizing granulomatous inflammation in the majority of patients. Eosinophilic infiltrates, panniculitis and vasculitis may be seen [31].

## 6. Conclusions

In conclusion, there are only minor differences in the histological features of genital dermatoses in children from adults.

Even with atypical findings of a genital naevus on biopsy, vulval melanocytic lesions in juveniles have a benign course. It is important to recognise this to avoid unnecessary wide excisions, which can cause psychological trauma and physical scarring in young children. Melanoma does not seem to occur in this cohort. In the setting, pathologists should particularly beware of overdiagnosis when interpreting findings in biopsies and excision specimens, and shouldn’t advise a diagnostic excision to avoid unnecessary and possibly mutilating procedures. If there is any diagnostic doubt, then referral to an expert centre should be recommended. Specific guidelines for the management of genital melanocytic lesions in children are needed.

## Data Availability

No new data were created or analyzed in this study.
